# Gold Nanoparticles: Multifaceted Roles in the Management of Autoimmune Disorders

**DOI:** 10.3390/biom11091289

**Published:** 2021-08-30

**Authors:** Khadijeh Koushki, Sanaz Keshavarz Shahbaz, Mohsen Keshavarz, Evgeny E. Bezsonov, Thozhukat Sathyapalan, Amirhossein Sahebkar

**Affiliations:** 1 Hepatitis Research Center, Lorestan University of Medical Sciences, Khorramabad 6813833946, Iran; koushki989@yahoo.com; 2 Cellular and Molecular Research Center, Research Institute for Prevention of Non-Communicable Disease, Qazvin University of Medical Sciences, Qazvin 3419759811, Iran; sanaz.ks_023@yahoo.com; 3 Department of Medical Virology, The Persian Gulf Tropical Medicine Research Center, The Persian Gulf Biomedical Sciences Research Institute, Bushehr University of Medical Sciences, Bushehr 7514763448, Iran; Keshavarz.m@bpums.ac.ir; 4 Laboratory of Cellular and Molecular Pathology of Cardiovascular System, Institute of Human Morphology, 3 Tsyurupa Street, 117418 Moscow, Russia; evgeny.bezsonov@gmail.com; 5 Laboratory of Angiopathology, Institute of General Pathology and Pathophysiology, 8 Baltiiskaya Street, 125315 Moscow, Russia; 6 Academic Diabetes, Endocrinology and Metabolism, Hull York Medical School, University of Hull, Hull HU32RW, UK; Thozhukat.Sathyapalan@hyms.ac.uk; 7 Applied Biomedical Research Center, Mashhad University of Medical Sciences, Mashhad 9177948564, Iran; 8 Biotechnology Research Center, Pharmaceutical Technology Institute, Mashhad University of Medical Sciences, Mashhad 9177948954, Iran; 9 School of Medicine, The University of Western Australia, Perth, WA 6009, Australia; 10 School of Pharmacy, Mashhad University of Medical Sciences, Mashhad 9177948954, Iran

**Keywords:** gold nanoparticles, autoimmune diseases, immunomodulatory effects, nanomedicine

## Abstract

Gold nanoparticles (GNPs) have been recently applied for various diagnostic and therapeutic purposes. The unique properties of these nanoparticles (NPs), such as relative ease of synthesis in various sizes, shapes and charges, stability, high drug-loading capacity and relative availability for modification accompanied by non-cytotoxicity and biocompatibility, make them an ideal field of research in bio-nanotechnology. Moreover, their potential to alleviate various inflammatory factors, nitrite species, and reactive oxygen production and the capacity to deliver therapeutic agents has attracted attention for further studies in inflammatory and autoimmune disorders. Furthermore, the characteristics of GNPs and surface modification can modulate their toxicity, biodistribution, biocompatibility, and effects. This review discusses in vitro and in vivo effects of GNPs and their functionalized forms in managing various autoimmune disorders (Ads) such as rheumatoid arthritis, type 1 diabetes, and multiple sclerosis.

## 1. Introduction

The immune system is a complex network including immune cells and molecules that protect against various infections and diseases. However, sometimes the immune system loses its self-tolerance following specific conditions. This leads to the immune system attacking and damaging multiple tissues in the body and eventually leading to autoimmune disorders (Ads). These diseases are characterized by the improper activation of excessive and pathological immune responses to self-antigens, a specific organ or tissue in the absence of any tissue or cell injury, toxins exposure, or microbial attack [1]. The prevalence of autoimmune diseases is increasing [2]. The most common ADs include systemic lupus erythematosus (SLE), type 1 diabetes (T1DM), rheumatoid arthritis, psoriasis, multiple sclerosis (MS), celiac disease (CD), and inflammatory bowel diseases (IBD).

Although the exact etiology of many autoimmune diseases remains unknown, most studies suggest that it has probably genetic factors combined with environmental and epigenetic factors resulting in the breakdown of immune system homeostasis, contributing to the development of autoimmune diseases [3,4]. Among the environmental factors, three significant socioeconomic status-related factors are assumed to drive these conditions, including infections, ecology, and nutrition [5]. Unfortunately, there has been a considerable increase in the global frequency and the prevalence of ADs in the West over the last decades. This is due to notable changes in lifestyle habits such as diet and eating habits, environmental and occupational exposure to a high degree of air pollutants, and infectious disease (ID) [6]. It is estimated that AD affects around 5–7% of the global population. The prevalence is rising globally, with an onset often during adulthood nearby 40–50 years of age. ADs also have a higher incidence in women compared with men [7,8]. According to the National Institute of Health (NIH) reports, about 23.5 million people in the USA suffered from ADs and are among the top 10 leading causes of mortality in females aged up to 64 years. In addition, approximately 10 percent of the adult population in European countries was diagnosed with AD in 2018 [2,9]. The economic and societal burden of ADs results in a high medical cost for providing patient care, and relapsing AD conditions often result in reduced quality of patient life. The estimated annual cost of patients treated with ADs is approximately 100 billion dollars. Moreover, standard treatments are not very sufficient and commonly cause various unwanted side effects [10].

The application of nanoparticles (NPs) for modulating the immune system is currently an attractive field. Increasingly, some metal nanoparticles, mainly gold nanoparticles (GNPs), are used to manage various medical conditions because of their unique properties. GNPs with unique optical and chemical properties are attractive metal nanoparticles in the nanoscience field. They are most frequently applied in different biomedical purposes due to their stability, biocompatibility, and inert nature. Additionally, studies have shown that GNPs exhibit anti-inflammatory and anti-oxidant properties. Therefore, they are characterized as active therapeutic agents to diagnose and treat different ADs such as T1D, RA, and IBD. Since some nanocomposites and nanomaterials are immunomodulatory or immunotoxic, a comprehensive review of the interactions between nanomaterials and the immune system would be greatly useful to the design of various research studies. This review aims to outline the interactions of immune systems with GNPs and drugs-based GNPs in managing different ADs. In this article, we discussed the toxicity, anti-inflammatory, and antioxidative potential of GNPs and subsequently selected and reviewed various investigations of GNP-based drugs and their potential as therapeutic applications in ADs.

## 2. Pathogenesis of Autoimmune Diseases

Autoimmune disease results from dysregulation of the immune system and losing tolerance, which leads to excessive and pathologic innate and adaptive immune responses against cellular or organ-specific self-antigens, subsequently resulting in tissue destruction and dysfunction through inducing inflammation and injury in affected systems [1]. The hyper-activation of the innate immune responses and failure in T- and B-cell repertoire selection or a failure to regulate activated T- and B-cells can trigger the initiation of autoimmunity in susceptible individuals. In addition, the presence of autoreactive B- and T-cells several years before the appearance of clinical diseases indicates that multiple triggers could act sequentially to promote disorders [4]. Autoimmune inflammations are directed by cognate interactions between antigen-presenting cells (APCs) and self-reactive T-cells [11]. In this regard, although immune tolerance is induced by presenting self-peptide–MHC (pMHC) complexes by APCs to non-inflammatory lymphocyte cells in steady-state, the recognition and engagement of pMHC by autoreactive lymphocyte cells in the context of inflammation lead to the activation of T- and B-cell and autoimmune disorders [12,13,14]. Therefore, the main purpose of autoimmune therapeutic strategies is to promote tolerance through targeting APC–autoreactive cells interaction. Furthermore, despite the essential role of dendritic cells (DCs) and maybe macrophages (MQs), especially in the initial activation of autoreactive T- and B-cells, evidence suggests cognate T/B-cell interactions are vital in this event. Some studies via elimination of T-cell populations using thymectomy and or anti-T-cell antibodies have shown the vital roles of cellular immunity in the development of some ADs marked by autoantibody production, and B-cell dysregulation is supposed to play a key role [15,16]. Eventually, T- or B-cell genetic knockouts revealed that both cell subsets are required to develop certain autoimmune diseases such as SLE [17,18].

Overall, these mechanisms and affected organs via autoimmunity are broadly varied. For example, in multi-organ ADs such as RA, inflammation is mainly localized to the joints following immune cell infiltration of the synovial membrane [19]. This category generally involves a strong autoantibody (Th2) component. However, in organ-specific cases, autoinflammatory responses occur against a specific organ such as T1DM, which destroys β-islet cells of the pancreas and eventually leads to insulin deficiency [20]. Similarly, autoimmune diseases of the gastrointestinal (GI) tract seen in IBDs subsets, including ulcerative colitis (UC) and Crohn’s disease (CD) [21], are generally T-cell-mediated (Th1 or Th17 subsets) processes [22]. Furthermore, some cases such as MS exhibit both systemic and organ-specific characteristics. For example, MS is characterized by inflammation in the brain and spinal cord, resulting in axon and myelin damage, which results in central nervous system disturbance [23].

## 3. The Role of Inflammation and Inflammatory Cytokines in ADs

Inflammation serves as a first line of physiological defense against tissue injury and infection. Unfortunately, these inflammatory responses continue in some chronic conditions and result in significant tissue or organ injury. These unusual and unregulated inflammatory responses are closely related to various autoimmune disorders, such as inflammatory bowel disease (IBD), rheumatoid arthritis (RA), diabetes, systemic lupus erythematosus (SLE), and gout [21,24,25,26]. However, the exact pathogenesis of inflammation in many autoimmune disorders has remained somewhat unknown. Therefore, increasing our knowledge of various regulating mechanisms of inflammation can lead to many remarkable clinical approaches to treating autoimmune diseases.

As mentioned before, inflammatory responses mediated by T-cells have been identified as having crucial roles in developing autoimmune disorders. Recently, many studies have shown that abnormal immune responses of T-cells, including Th1, Th2, Th17, Th22, and T-Reg cell responses, have a pivotal role in developing inflammation in autoimmune disorders [27]. The enhanced activity of CD4+ T-helper lymphocytes is one of the most common pro-inflammatory phenotypes in ADs. Three major types of CD4+ T-helper subsets are significant in this respect: Th1, Th2, and Th17 cells. As a general rule, the cell-mediated processes and dominant Th1 and their cytokines such as IL-2 and interferon-gamma (INF-γ) are involved in organ-specific ADs [28,29]. In contrast, severe humoral (antibody-based) responses and elevated Th2 cytokines levels such as IL-4, IL-5, and IL-10 are typically related to multisystem ADs [28,29]. Additionally, the Th2 responses usually lead to the exacerbation of fibrosis in many ADs by higher induction of macrophages [30]. In addition, the Th17, via producing IL-23 as a neutrophil chemotactic and activating factor phenotype, contributes to the genesis of ADs [31]. Generally, both Th17 and Th1 cells are generated parallel based on overlapping only partly in their functions [32,33]. Significantly, Th17 cells can direct ADs in the absence of a concomitant Th1 immune response [34].

In addition to T-cells, various other immune cells including B-cells are also involved in developing autoimmune diseases. The different B-cell subsets play multifaceted roles in autoimmune diseases [35]. It has been shown that the B-cells, often via the production of antibodies, play a deleterious role in developing autoimmune diseases [36]. Additionally, regulatory B-cells (Bregs) have received a great deal of attention for suppressing inflammation by repressing differentiation of the Th1 and Th17 immune responses in the development of autoimmune disorders [37]. It is important to state that B-cell subtypes and their antibody production and mechanisms are very diverse. The characteristics and effects of autoantibody-secreting plasma cells depend on their tissue localization. On the other hand, innate immune cells such as monocyte and macrophage cells have been considered major regulators in the inflammation of the liver and other organs. A study by H. Li et al. demonstrated that M1-polarized macrophages could promote generating hepatic progenitor cells (HPC) with the self-renewing phenotype, which is associated with activation of the Notch signaling HPCs in primary sclerosing cholangitis [38]. Toll-like receptors (TLRs) are a group of pattern recognition receptors (PRRs) that play a crucial role in the innate immune response [39], which by activating the innate immune cells play a key role in developing autoimmune diseases. APCs, particularly DCs, are crucially involved in autoimmunity disorders through their capability in priming and activation of autoreactive T-cells and subsequently breaking immune tolerance [40]. Moreover, it has been shown that human amnion mesenchymal cells (hAMC) could be promising cells for MS therapy via reducing inflammation and developing remyelination in EAE models [41].

## 4. Clinical Management of Autoimmune Disorders

There is a lack of sufficient knowledge about the etiologies of different ADs. Therefore, the current clinically relevant treatments are focused on symptom management and control of disease to reduce the number of relapsing events [42,43,44] until the detection of effective targeted therapies. For example, the application of insulin for T1DM patients to maintain blood glucose homeostasis [42,43,45] or the use of tear and saliva replacements as the main treatment of Sjögren’s syndrome (SS) patients, accompanied by supplemental medications to address additional complications. Recently, biological therapies such as proteins and antibodies have been developed as an alternative option to treating ADs, such as modulating the expression of tumor necrosis factor-alpha (TNF-α) used to manage patients with IBDs [46,47]. Interferon-beta (IFN-β) also is widely used in the management of MS [48]. However, these biological drugs are only effective in some of the patients [49]. As a result, the next generation of ADs therapies should focus on precise medical approaches to dampen disease-propagating agents’ effects rather than relieve the symptoms as they arise.

Recently, NPs have shown many advantages in treating inflammatory and autoimmune diseases, which offer some innovative strategies to improve the therapeutic efficacy compared to traditional therapies. Numerous studies have indicated that NP-based drug delivery could enhance the treatment efficacy in comparison to current therapies through more specific targeting of infected tissues than normal, which significantly reduces prescribed drug dosage and reduces drug adverse side effects. The NPs also could improve the bioavailability and pharmacokinetics of therapeutic agents. Furthermore, the functionalization of the NP surface with diverse immunomodulators and antigens can optimize their functions by delivering coordinated messages into the immune system. Therefore, NP-based therapies are promising agents to transform a multifaceted program for the pharmaceutical industry.

## 5. Nanotechnology and Autoimmune Diseases

Nanotechnology is one of the most exciting 21st-century industrial innovations, which applies to various industrial products and medical purposes such as therapeutic, drug delivery, and diagnostic applications. The nanoparticles are tiny materials with sizes ranging between 1–100 nm. They can be categorized into different classes based on their composition: organic (e.g., lipid, polysaccharide, a polymeric matrix), inorganic (e.g., gold, silver, carbon), and liposomes. The various applications of these particles are rapidly growing due to their chemical, electronic, and optical properties [50]. Indeed, NPs, by carefully altering their chemistry, size, and proper functionalization with various targeting agents, could carry therapeutic agents to specific and unavailable tissues or cells [51,52]. Furthermore, these vehicles can also protect therapeutic agents from degradation by enzymes and invasion by host defense until reaching the desired sites.

Recently, nanoparticle engineering has become an exciting and emerging field for targeting the immune system. However, the physicochemical properties of NPs are significantly different compared to their conventional materials. Thus, their properties in the immune system can be a “double-edged sword” with positive or negative effects on health upon exposure. Collectively, in the engineering of nanomaterials for in vivo conditions, three vital immune-related outcomes must be considered, including (1) immune-mediated destruction, which initiates a defensive immune response and eventually leads to the elimination of nanoparticles; (2) immunotoxicity that could cause immune system damage; and (3 immunocompatibility that does not interfere with the normal immune responses [53]. In addition, some nanoparticle properties, including hydrophilicity/hydrophobicity, charge, size, and nanoparticle coatings’ steric effects, determine toxicity and adaptability of NPs with the immune system [54,55]. Additionally, the toxicity of some metal-based nanoparticles such as gold, copper, and silver might enhance by decreasing their diameter [56].

In recent years, the use of nano drugs in the treatment of autoimmune disorders has been expanded, which presents various advantages in comparing traditional therapies, including (1) facilitating the cross-capability of the drug over biological barriers such as the blood–brain barrier; (2) the combination of a diagnostic tool with a therapeutic agent, known as “theranostic” agents and leads to increasing the drug specificity, reducing the side effects and overcoming multidrug resistance mechanisms; (3) improving the half-life of drugs unique biomolecules and peptide-drugs by preserving them from enzymatic hydrolysis during the circulation and the environment; (4) increasing the delivery of poorly water-soluble drugs and their treatment efficacy; (5) the release of the therapeutic agent over a period of time; and (6) enhanced bioavailability and pharmacokinetics of drugs. Therefore, NP-based therapies have great potential to improve treatment efficacy in various disorders and could transform into a multifaceted platform for the pharmaceutical industry. In the past few years, several nano-drugs were approved by the FDA and are already in the market, and many are currently under clinical trials [57]. On the other hand, inorganic and organic nanomaterials could be captured by the reticuloendothelial system cells according to their surface modification, shape, and size. Therefore, these NPs could be a passive targeting system that preferentially delivers to innate immune cells, including APCs, dendritic cells, macrophages, neutrophils, and other immune cells. This NPs affinity toward the phagocytic cells represents potential applications of NPs, as immuno-therapeutic tools, to apply targeted therapies in immune disorders involving circulating and localized immune cells such as inflammatory and autoimmune disorders. Therefore, the design of high-quality NPs could develop a promising next-generation of nano drugs to manage various inflammatory and autoimmune conditions [58].

The applications of nanoparticles for inducing antigen-specific tolerance in autoimmune disorders are being increasingly developed. As mentioned above, the interactions between the antigen-presenting cells (APC) and autoantigen-specific T-cells play a vital role in developing autoimmune diseases. Therefore, it is not unexpected that many therapeutic strategies against autoimmune diseases aim to develop tolerance via targeting the APC–T-cell interaction. In the last decade, various strategies have been applied to inducing immune tolerance to many antigens. Autoimmunity is often accompanied by an inflammatory milieu that induces autoreactive T- and memory B-cells. Inflammation leads to the activation of APCs such as DCs and MQ, subsequently presenting the autoantigens to activate acquired immune cells. Therefore, the targeting of these cells via the application of NPs could significantly impact our ability to influence autoimmunity development. Therefore, two main strategies for inducing immune tolerance using the NPs application include targeting self-reactive lymphocyte cells and the APCs [59].

The phagocytic cells such as APCs are natural candidates for evaluation as tolerogenic tools due to their ability to uptake nanoparticles. Notably, we could specifically change the NPs effect on various immune cell populations by changing their properties such as shape, size, charge, and administration route [60]. Furthermore, these approaches could block the autoreactive process and ultimately affect adaptive lymphocyte functionality, resulting in the deletion, exhaustion, and induction of Breg and Treg cells [61]. Nevertheless, the application of NPs in autoimmune disease treatment via inducing antigen-specific tolerance remains largely unexplored.

## 6. Gold Nanoparticles, Characterization, and Immune Stimulation

Gold nanoparticles (GNPs) are attractive NPs with unique physical, chemical, and optical properties in nanotechnology and bio-nanotechnology fields. Due to many advantageous and unique properties of GNPs, such as easy and controllable fabrication, high stability, oxidation resistance, water-solubility, plasmon resonance, high surface reactivity, high drug-loading capacity, low cytotoxicity, and cost–benefits, they have been widely investigated and have shown potential applications in various engineering, chemistry, and biomedical fields [62,63]. In particularly, GNPs have become one of the ideal metal nanomaterials for medical purposes because of their biocompatibility and inert nature. In addition, GNPs offers other therapeutic options than traditional gold salts, such as radiotherapy enhancement due to the preferential absorption of x-rays via high-atomic-number GNPs [64].

Apart from numerous advantages, easily functionalization of GNPs with different molecules can offer entirely new therapeutic options for combination therapy [64] and also combined therapy and diagnostics (theranostics). It has been demonstrated that GNPs are an efficient carrier with a controllable release for diverse therapeutic agents such as antineoplastic medications [65], antioxidants [66], antibiotics [67], proteins [68,69], nucleic acids [70], and glucose [71]. The nanospheres and nanorods shapes of GNPs are the most-favored gold structures that have been investigated for biomedical purposes due to their well-characterized synthesis. In addition, GNPs offer other therapeutic options than traditional gold salts, such as radiotherapy enhancement due to the preferential absorption of x-rays via high-atomic-number GNPs [64]. Therefore, the GNPs’ functionalization with various therapeutic and targeting led to further growth of their applications. Wide successful usage of GNPs in many biomedical fields such as diagnostics [72], therapeutics and vaccine development [59,72], drug, gene delivery and imaging [70,73], and electrochemical biosensors [74] has been obtained through controlling the size and shape of these particles via using proper synthesis as well as modifying through suitable functionalizing groups.

The continuous rise in utilization of GNPs has increased considerations of the safety and toxicity of these NPs for possible toxicological effects. It has been evident that the cytotoxic effects of GNPs are nearly zero or at least significantly less than other NPs such as silver NPs [75]. Several studies have demonstrated that GNPs are nontoxic to mice and humans [76,77]. Non-toxic effects of 12.5 nm GNPs have been shown in the lungs, liver, spleen, kidneys, or brain [78]. However, an in vitro study showed a size-dependent toxic impact of GNP has occurred for 1.4 nm GNP but not for 15 or 0.8 nm GNP [79]. Another study in human cells has shown that GNPs are non-toxic in 250 nanometer sizes and rejected their toxicity. It has been indicated that GNPs’ toxicity increased by decreasing the diameter of NPs [56]. Chen et al. showed that GNPs with 3, 5, 50, and 100 nm (8 mg/kg/week, for three weeks) did not show any cytotoxicity and harmful effects, while 8–37 nm GNPs showed cytotoxicity effects. Interestingly, they indicated that the surface modification of the GNPs with peptides significantly ameliorated this toxicity and was associated with their ability to induce an antibody response [80]. A subsequent study in the same condition by Reeves et al. [78] showed that different doses (40, 200, and 400 μg/kg/day, for eight days) of 12.5 nm diameter GNPs did not produce any toxicity and side effects in various organs of mice. In line with this finding, another study has shown that functionalized GNPs with 13–20 nanometer diameters do not cause acute side effects [81]. Therefore, it is supposed that dosage and surface modification of GNPs are other important factors of GNPs’ cytotoxicity.

In addition to size and shape, surface modification of NPs is another important factor that can impact the biocompatibility and toxicity of GNPs, which are still being explored. It has been shown that 20 nm diameter gold nanospheres covered with mercaptopropane sulfonate have nontoxic effects on the human keratinocyte cell line, but 16.7 and 43.8 nm diameter gold nanorods functionalized with PEG could induce ROS production significantly and upregulate expression levels of genes related to cellular stress and toxicity proposing. Therefore, it seems that GNP modifications play a crucial role in GNP-mediated cellular response [82]. On the other hand, various results demonstrate that tissue distribution and accumulation of GNPs is size-dependent, and the smallest nanoparticles show the most widespread organ distribution [83,84]. For example, it has recently been shown that 50 nm diameter GNPs are more permeable to cells and more effectively accumulate into the tumor after a single IV administration. Conversely, larger GNPs were fundamentally concentrated in and around blood vessels and the periphery of the spherical tumor, preventing their deep penetration into tumors [85]. Additionally, biodistribution studies reported that PEG-GNPs with 5 and 10 nm sizes accumulate in the liver, and 30 nm diameter GNPs accumulated in the spleen, while 60 nm diameter GNPs did not significantly accumulate in mice organs. These data suggest that the toxicity of PEG-GNPs is complicated, and it cannot be concluded that smaller particles have more significant toxicities and vice versa [86]. Besides size and shape, surface modification of NPs is another important factor that can impact the biocompatibility and toxicity of GNPs. It has been reported that although 20 nm diameter gold nanospheres covered with mercaptopropane sulfonate have nontoxic effects in the human keratinocyte cell line, 16.7 and 43.8 nm diameter gold nanorods functionalized with PEG could significantly induce ROS production and upregulate expression levels of genes related to cellular stress and toxicity proposing. Therefore, it seems that GNPs modifications play a crucial role in GNP-mediated cellular response [82]. Moyano et al. [54] demonstrated the importance of hydrophobicity of engineered GNPs in immune system activation in vitro and in vivo.

Collectively, the main determinative characteristics of GNPs, including size, shape, chemical composition, surface properties and modifications, and environmental impact, could modify biodistribution, cytotoxicity, and biocompatibility of GNPs, which still need to be further evaluated. It has to be noted that although the zerovalent of GNPs can be a valuable alternative, replacing the potential of metallic gold [87], their intracellular uptake and subsequent responses could vary according to their particle characterization. Hence, a detailed assessment of the interaction between GNPs or GNP–biomolecule conjugates and the immune system can be crucial for estimating both unintended and intended effects of their applications.

## 7. Gold Nanoparticles for Immune Stimulation; Focus on Anti-Inflammatory and Antioxidant Properties

**Figure 1:**
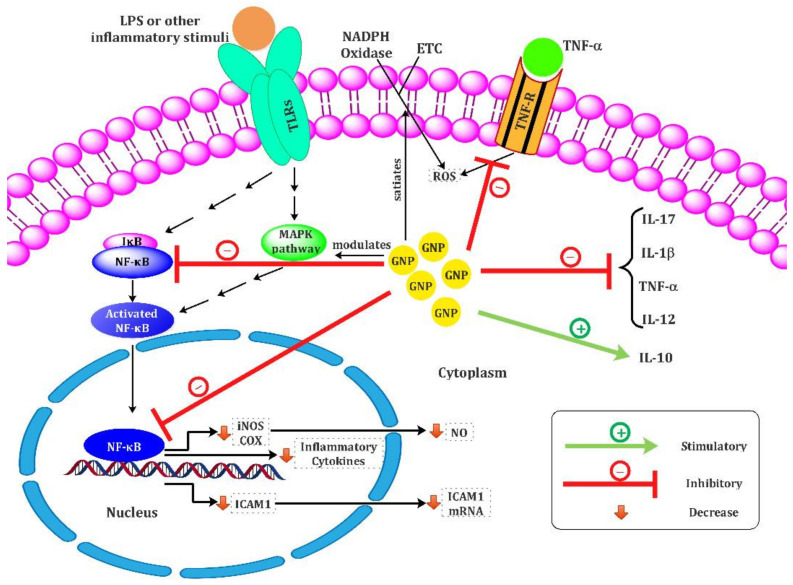
The adopted mechanisms for anti-inflammatory effects of GNPs include; (1) modulation of MAPK and PI3K pathways in Kupffer cells (liver macrophages) and hepatic cells, and the MAPK pathway, a key mechanism of inflammatory signal transduction from the cell surface to the nucleus, which leads to activate transcription factors and alterations in gene expression following LPS binding to TLRs. The PI3K pathway is involved in gene expression, protein synthesis, cell proliferation, and cytokine stimulation. GNPs negatively regulate Kupffer and hepatic satellite cells’ activity and affect their pro-inflammatory cytokine profile and oxidative stress via the modulation of AKT/PI3K and MAPK signaling pathways. In addition, the GNP treatments could reduce the activation of NF-κB through ERK1/2MAPK/Akt/tuberin-mTOR pathway-mediated targeted inflammatory gene expression and cellular stress responses. (2) Significant inhibiting the production of several pro-inflammatory cytokines such as LPS-triggered TNF-α, IL-1β, and IL-17, which can downregulate IL-1β-induced epithelial cells proliferation. Moreover, GNPs could decrease the raised level of IL-12 production, which could leads to a change in the cell-mediated immune response of pro-inflammatory response (TH1) to anti-inflammatory response (TH2). (3) Reducing ROS production; the reactive oxygen species (ROS) are oxygen metabolites (OH^−^, O2^−^, H_2_O_2_) that have potent oxidizing features and can oxidize proteins and lipids in the cells and causes DNA damage. Reactive nitrogen species (RNS) are a combination of superoxide anion (O2^−^) and NO, which induce nitrosative stress and promotes the production of ROS. GNPs could lead to satiating and downregulating the phagocyte-produced ROS in a dose-dependent manner. Therefore, GNPs act as potential anti-oxidant and anti-inflammatory agents.

Anti-inflammatory and antioxidant effects of GNPs have been investigated in various in vitro and in vivo studies. Numerous studies have shown that GNPs have potential anti-inflammatory and antioxidant effects and lead to the downregulation of cellular responses, induced proinflammatory cytokines, and oxidant mediators. The mechanisms employed by the GNPs are shown in Figure 1. In this section, we discuss various studies to evaluate the immunomodulatory effects of GNPs on macrophages and other immune cells, their functions, and cytokine production such as TNF-α and IL-1β, IL-6, IL-10, and IL-17.

Some studies indicated that the immunomodulatory effects of GNPs were exerted by their capability in inhibiting the expression of the nuclear factor kappa B (NF-κB) transcription factors and downregulating subsequent cellular responses and cytokines production. For example, a study by Jeon et al. [88] indicated that GNPs could block the activation of NF-kB by interacting with Cys-179 of the IKKβ subunit, which results in the suppression of the production of subsequent pro-inflammatory cytokines such as IL-1β and TNF-α. Furthermore, Rizwan et al. showed that GNPs decreased the activation of NF-κB signaling through interfering effects on ERK1/2MAPK/Akt/tuberin-mTOR pathways and subsequent inflammatory and cellular stress responses, which eventually lead to modulation antioxidant defense mechanisms and enhancing anti-inflammatory agents [89]. Moreover, significant downregulating of mRNA expression of NF-kB, TNF-a, COX-2, and iNOS were reported for 20 nm GNPs in the collagen-induced arthritic (CIA) rat model [90].

Dohnert et al. showed that 10 nm GNPs have anti-inflammatory effects through a significant decrease in inflammatory parameters, including IL-1β and TNF-α, in a rat model of tendinopathy [91]. In line with these data, Edrees et al. also showed that GNPs have an anti-inflammatory role by significantly reducing TNF-α, IL-6, and CRP (*P* < 0.001) in diabetic rats [92].The first in vitro and in vivo evidence for the anti-inflammatory effects of GNPs in human vein endothelial cells (VECs) and its protective effect on vascular injury reported by Lai et al. [93] that has shown GNPs could decrease NF-κB signaling pathways, TNF-α production, and subsequent TNF-α-induced intracellular ROS and CAM protein production. The data showed that GNPs via two different pathways suppressed CAM protein, including increasing CAM ubiquitination and degradation and interference in NF-κB signaling pathways, which reduces rat arterial neointima formation by the attenuation of monocyte adhesion VECs.

On the other hand, some studies indicated the involvement of anti-inflammatory cytokines in the GNPs’ immunomodulatory effects. In this regard, Chen et al. have shown an increase in anti-inflammatory cytokine IL-10 secretion from macrophages in a dose-dependent manner of GNPs in the hepatic injury rat model [94]. These data are in line with our observation that showed 15 nm diameter GNPs and GNPs–allergen proteins conjugates increased anti-inflammatory IL-10 and TGF-b cytokines [68,69] and decreased IL-17 levels [68] in allergic inflammation conditions. In addition, a study by Kingston et al. showed that 50 nm diameter GNPs did not affect macrophage viability and inflammatory cytokines alone but significantly decreased LPS-driven inflammatory responses include inflammatory cytokines such as TNFα and IL-17, and TH17 responses, and ROS production in a dose-dependent manner. These results suggested that GNPs do not have a cytotoxic effect on immune cells but could affect cellular responses to inflammation or infection via changing the cytokines balance [95]. Another study by Koushki et al. showed 15 nm diameter GNPs significantly decreased IL-17 levels in allergic inflammation conditions [68]. For the first time, Sumbayev et al. indicated a size-dependent anti-inflammatory activity of GNPs by downregulating IL-1β-induced cellular responses both in vitro and in vivo. The finding showed 5 nm GNPs completely inhibited the inflammatory process, while 15 nm diameter GNPs had moderate effectiveness, and 35 diameter nm GNP did not display any statistically significant effect [96].

On the other hand, various studies reported the potential antioxidant effects of GNPs. It has been indicated that GNPs could suppress reactive oxygen species (ROS], such as superoxide anion radicals (O2^−^) and H2O2, in a dose-dependent manner [97]. Carvalho et al. also showed that 7.4 nm diameter GNPs lead to a reduction in biochemical markers of liver injury, oxidative stress SOD-1 and GPx-1, pro-inflammatory cytokines such as IL 1β, and TNF-α via modulation of AKT/PI3K and MAPK signaling pathways in a liver injury rat model compared to control groups [98]. In addition, in vivo experiments on animal models of inflammatory conditions showed the antioxidant and anti-inflammatory features of the GNPs via a reduction in oxidative tissue damage markers and pro-inflammatory cytokines such as TNF-α, IL-1β, and IL-6. [96,99,100,101].

Although the relation between GNPs and oxidative stress has not yet been well-established, Zhou et al. [102] reported the highest antioxidant effects of 9 nm diameter GNP, which could mimic antioxidant enzyme action through direct interaction with hydroxyl radicals and superoxide anions to form less-reactive side products. In line with data, some research demonstrated that GNPs directly bind and neutralize free radicals, especially the superoxide anion; this effect depended on the size, surface of the molecule, and GNP dosage [102,103]. Moreover, another study showed similar dose-dependent properties of GNP as a free radical-chelating agent [104]. Various studies have shown that GNP therapy can decrease superoxide and nitrite levels in animal models without other supplementary therapies [105,106]. In particular, a study by Ma et al. reported that GNPs reduced nitric oxide-induced synthase (iNOS) gene expression and NO production by blocking the activation of NF-κB and STAT1 in lipopolysaccharide (LPS)-stimulated RAW264.7 cells [107]. Sul et al.’s results indicate that GNPs suppress RANKL-induced osteoclast formation in vitro by reducing ROS production and upregulating the antioxidant enzyme Gpx-1 level upon stimulation of BMMs. Therefore, GNPs reduce oxidative stress during inflammatory processes without any cytotoxicity effect on the BMMs upon these conditions [108]. GNPs also suppressed matrix metalloproteinase-8 (MMP-8) and MMP-9 activity in the RAW264 cell line, without any cytotoxicity effect in different concentrations [109].

Some studies introduced GNPs as an antioxidant agent that could improve enzymatic antioxidant defenses and suppress the cascade contributing to ROS formation [110]. Moreover, it has been reported that 20 nm GNPs significantly increase the reduced activities of superoxide dismutase (SOD) and catalase (CAT) in Mdx-treated mice. Therefore, GNPs exert their antioxidant activity by decreasing nitrogen species and ROS production, improving antioxidant activity, and subsequently reducing oxidative damage, inflammation, and pro-apoptotic proteins [111]. Furthermore, both antioxidant and anti-inflammatory effects of GNPs have been shown by a decreased expression of CD68, a membrane protein of macrophage, and an increased expression of SOD in an epithelial lesion model [112].

Despite many studies that indicate the anti-inflammatory and antioxidant effects of GNPs, contradictory results have been reported concerning the immunomodulatory effects of GNPs by some studies. Some literature stated GNPs are toxic to MQ and can trigger an inflammatory response [81,113] and cause oxidative stress [114,115]. Previous studies have shown that various GNP characteristics and their surface modifications can affect their immunomodulatory activity. Therefore, understanding how GNPs affect or modulate the immune system is pivotal to better understanding the potential risks in developing novel nanoplatforms for therapeutic applications. Khan et al. observed that both 10 and 50 nm diameter GNPs significantly enhanced transient gene expression of inflammatory cytokines (IL-1𝛽, IL-6, and TNF-𝛼) in rat liver cells on day 1, which subsequently subsided following sub-chronic treatment on day 5. In addition, 50 nm GNPs induced more severe inflammation than 10 nm GNPs; these results proposed a biocompatibility potential of GNPs with a medium size in a time-dependent manner [81]. These results showed that GNPs with a medium size (10–50 nm) have biocompatibility. They result in a transient increase in pro-inflammatory cytokines followed by their normalization during sub-chronic repeated exposure.

Moreover, it reported that GNPs with about 5 nm diameter size upregulated the expressions of pro-inflammatory cytokines more than silver-NPs (AgNPs). Therefore, it was supposed that adsorption of serum protein onto the GNPs surface, via GNPs negative charge, led easily to endocytosis and a subsequent high level of immunological reaction and cytotoxicity of GNPs than AgNPs. However, the data showed a time- and dose-dependent influence of GNPs on splenocytes. The lowest dose had a pro-inflammatory effect and stimulated the synthesis of proinflammatory cytokines including IL-1β, IL-6, and TNF-α [116]. However, some studies reported contradictory results [75,117]. For example, Nishanth et al. [117] indicated that although various NPs including silver (Ag), aluminium (Al), carbon-coated silver (CAg), and carbon black (CB) nanoparticles resulted in inducing significant inflammatory and oxidant mediators such as TNF-a, IL-6, and COX-2 in macrophages through NF-kB and ROS signaling pathways, GNPs (20 and 40 nm) did not show any the NF-kB activation, IL-6 release, or ROS generation. These data support other reports on GNPs being not acutely cytotoxic. Therefore, it is supposed that different immunostimulatory effects are due to their different sizes and concentrations. Moreover, intratracheal instillation of both agglomerated and single forms of 50 and 250 nm GNPs resulted in a mild inflammatory reaction via a small increase in inflammatory cells, pro-inflammatory cytokines, and acute-phase proteins in a rat model. These effects were lowest for 50 nm GNPs.

Therefore, the finding indicated particle characterizations including size, concentration, and purity are significant characteristics to check since these features may vary from the manufacturer’s description [118]. Other studies also explained an association of the importance of the GNPs size and specific organ in enhanced expression of proinflammatory cytokines [119,120]. The size and shape of GNPs appear to have a significant role in uptake by immune cells, even by enhancing uptake of the serum proteins. Therefore, the difference in these parameters results in a large difference in the uptake by immune cells. For example, 74 × 14 nm rod-shaped NPs have lower cell uptake than 74 or 14 nm spherical nanoparticles, so results indicate that the immunoactivity of GNPs strongly depends on their physicochemical properties [121]. Therefore, responses to GNPs vary according to their shape, size, surface charge, capping agent, animal model, administration route, duration, and exposure frequency [122,123]. Further studies are needed to investigate the in vivo effects of surface-modified and bare GNPs on inflammatory and oxidant pathways. Numerous studies evaluated the GNPs effects on the inflammatory and oxidant parameters, listed in Table 1.

## 8. Impacts of Surface Modification of GNPs on Immune Response

According to the mechanisms mentioned earlier, both the core and coating agents of GNPs can impact the GNP effects [133]. The surface coating of nanoparticles is a key factor influencing their uptake and modulating the immune response. Interestingly, besides stabilizing attached biomolecules to the GNP surface, GNPs also improve the attached biomolecules’ uptake by immune cells such as macrophages, which plays an essential role in modulating the immune response, even for unrecognizable proteins by the macrophages. Therefore, the nanoparticle–biomolecule interaction is a reciprocal process in which both sides influence each other.

Bastús et al. [134,135] reported that the surface modification of GNPs with peptides, including amyloid growth inhibitory peptide (AGIP) or sweet arrow peptide (SAP), results in their recognition with the TLR-4 receptors and entry into the macrophages and subsequently inhibits macrophage proliferation and induces NO synthase and proinflammatory cytokines such as IL-1β, IL-6, and TNF-α. Whereas the macrophages could not identify either peptide or GNPs alone, they did not impact the production of nitric oxide (NO) and proinflammatory cytokines. On the other hand, it has been shown that GNP–peptide conjugates could be diffused widely throughout the epidermis and dermis, be uptake extensive by Langerhans cells and DCs, and eventually reduce the capacity of DCs to activate naive T-cells, thus adopting a regulatory rather than inflammatory phenotype [136]. In this regard, recent studies showed that functionalized GNPs with allergen proteins enhanced immunotherapy efficacy by decreasing inflammatory cytokine while increasing levels of anti-inflammatory cytokines compared to allergen protein. It was supposed that GNPs enhance protein absorption into administration sites such as skin and oral mucosa, which could lead to increased uptake and internalization of protein by DCs, and subsequent controlled release of allergen induce tolerogenic DCs in rhinitis allergic models compared to soluble allergen proteins [68,69]. Moreover, it has been indicated that GNPs conjugated with aptamers enhanced the therapeutic efficiency by inducing stability, specificity, and uptake of biomolecules. The obtained data of these studies showed that multi-functionalized GNPs with allergen protein and DC-specific aptamers significantly improve immunotherapy efficacy compared to both free protein and attached to GNPs, protein–GNPs conjugate, by significantly decreasing the pro-inflammatory cytokines (Il-1β, Il-17, IFN-γ) and inflammatory cell infiltrations, while significantly increasing anti-inflammatory (IL-10 and TGF- β) cytokines [68,69]. Interestingly, another study by Kalmodia et al. [66] indicated that functionalized GNPs with antioxidant peptides (Pep-A) synergistically improved the radical scavenging properties and enhanced antioxidant capacity compared to Pep-A and GNPs alone. This synergistic antioxidant effect could be due to the presence of polyphenols on the GNPs and the presence of antioxidant peptides. The data suggested the Pep-A and GNPs conjugate as a promising nano biocomposite for ROS scavenging activity, targeting cancer cells and inducing their apoptosis, minimizing the side effects resulting from chemotherapy, which might enhance the degree of success during treatment.

Moreover, some studies showed that the modification on GNPs surface with biomedical and proteins could be a strategy for activating and polarizing macrophages toward the antitumor direction. Therefore, this method was applied for vaccination experiments. It has been shown that among GNPs with poly (ethylene glycol) (PEG) modification or chicken ovalbumin (OVA) with sizes 12, 35, and 60 nm, larger particles or those conjugated with OVA were more easily phagocytized by macrophages with significantly more significant amounts. In addition, the OVA-coated GNPs induced higher production of IL-1β, TNF-α, and IL-6, while the PEG coating did not induce a significant inflammatory response, especially in larger sizes than 35 nm. Collectively, smaller GNPs induced stronger inflammatory reactions regardless of different kinds of surface molecules [137]. Mucin 1 (MUC1) is a membrane-tethered glycoprotein, which expresses on glandular epithelia and epithelial tumors, but tumor MUC1 differs from normal MUC1 by modified glycan side chains. MUC1 with modified glycan side chains can serve as a tumor-associated antigen to elicit MUC1-specific tumor immunotherapy and serve as a valid target for immunotherapy. It has been proven that MUC-1-protein-functionalized GNPs serve as a powerful macrophage activator, promoting the release of TNF-α, IL-6, IL-10, and IL-12 on peritoneal macrophages, resulting in predominant M1 polarization, which showed a promising prospect as a tumor vaccine [138]. Collectively, these data indicate a remarkable role of the surface coating of GNPs in cellular uptake and subsequent immune responses. This ability of GNPs to influence the regulation of macrophages and DCs activity can serve as a basis for new vaccine adjuvants. However, another study showed that M2 macrophages are more prone to take up and phagocytosis of PEGylated GNPs (15, 60, and 100 nm) than M1 macrophages, and both pre-and post-treatment with PEGylated GNPs inhibit the polarization of LPS-stimulated macrophages, especially for those pre-treated groups by larger GNPs (especial 100 nm PEGylated GNPs) [139]. It has been shown that GNPs affect cytokine production depending on their surface charge along with the type of surface-bound peptides but also in a cell-type-dependent manner. Bartneck et al. reported that the surface charge of GNPs mainly influences their uptake. At the same time, surface-coupled peptide sequences can alter cell functions, including the activation profile of DC, and modulate cytokine release in both DC and MΦ in a cell-specific manner [140].

Therefore, both GNPs and immune cells reciprocally influence each other. In this respect, some physicochemical parameters of GNPs such as size, shape, and surface charge and modifications showed an obvious impact on exocytosis, endocytosis, and polarization immune cells. In contrast, different immune cells or macrophage phenotypes, in turn, can affect the cell uptake and efflux of GNPs. Therefore, based on the desired purpose for upregulation or down-regulation of immune responses, the main characteristic of GNPs, type of surface coatings and protein corona formation, and target cells are important, which should carefully be considered to design efficient GNPs platforms for therapeutic or diagnostic applications. In this respect, GNPs were applied in various studies for clinical purposes, which are discussed in the following section.

## 9. GNPs Engineering for Therapeutic Application and Drug Delivery in Biomedical Use

Moreover, direct attaching therapeutic agents, drugs, and their derivatives onto the GNPs surface and co-loaded with other functional molecules has attracted wide interest, providing more active sites in favor of creating nano-drugs. It has been shown that DNA-conjugated GNPs emerged as an important class of nanomaterials with many attractive properties for bio-diagnosis and therapeutic applications, which showed efficient uptake and by about 50 different cell lines and induced significant cellular responses [141,142,143]. Jensen et al. [144] reported that functionalization of GNPs with small interfering RNAs (RNAi) could be an efficient nanosystem platform that effectively crossed the BBB (blood–brain barrier)/BTB (blood–tumor barrier) in Glioblastoma multiforme (GBM), entered tumor parenchyma, induced silence genetic lesions of GBM in vivo and in vitro, and efficiently reduced the tumor burden. The data suggested the GNPs-platform nanosystems as a promising approach for RNAi delivery for neutralizing various genes such as oncogenes, which could overcome major challenges associated with RNAi-based therapy and CNS-directed drug delivery. Song et al. [70] have reported a novel pH-responsive DNA-GNPs drug nanocarrier with efficient and rapid drug release into the target site, the pH-triggered drug release. They suggested this conjugate could be suitable for effective cancer chemotherapy at the cellular level. Moreover, the data showed PEGylation of GNPs-nanocarrier significantly enhanced its resistance to adsorption of non-specific serum protein. Therefore, they could induce high cytotoxicity via the efficient delivery of drugs to cancer cells.

Moreover, Chen et al. [145] designed a multifunctional nanosystem based on GNPs (GNP@CD-AD-DOX/RGD) for targeting chemotherapy of cancer, which is composed of three agents: cyclodextrin-modified GNPs, AD-Hyd- Doxorubicin (DOX) as an anticancer prodrug, and also AD-PEG8-GRGDS as a cancer-targeted peptide. The data showed that after uptaking the nanosystem by cancer cells, DOX rapidly released in response to pH resulting from the acid microenvironment of endo-lysosomes, and leading to inducing cancer cell apoptosis. Therefore, multifunctional GNPs have been suggested as ideal drug nanocarriers for enhancing anticancer efficacy due to their active targeting ability, promoting cellular uptake, and intracellular controlled drug release, which resulted in enhanced drug efficacy accompanied by reducing its side effects. In line with these data, some studies [146,147] have shown that co-functionalized GNPs with receptor-targeting agents and also drugs enhance their cellular uptake efficiency via binding the conjugates to membrane receptors and subsequent receptor clustering, and also increasing drug release and delivery, which resulted in enhancing the efficacy of GNP–drug conjugates for active-targeting therapies in cancers. It has been reported that the conjugation of methotrexate (MTX) onto GNPs’ surfaces represented a promising efficient, targeted chemo-photothermal therapy for RA, which significantly enhance intracellular releasing of MTX at photo-thermal temperature (42 °C) and acidic pH conditions. The data showed that combining methotrexate and GNPs resulted in pronounced anti-inflammatory effects by reducing the production of IL-6, TNF-a, and IL-1b by monocytes and macrophages and improved RA compared to MTX and GNPs treatments alone [148]. Farooq et al. [149] suggested PEG-GNPs as efficient nanocarriers for combined therapy in cancers. The data showed PEG-GNPs coated with DOX or bleomycin (BLM) strongly enhanced their therapeutic efficacy at lower doses via the active targeting and delivery to HeLa cells, reducing the drug’s systemic toxicity while maintaining its cytotoxic activity. Moreover, both the acidic environment and laser light could induce the further release of drugs.

Nosratabadi et al. [150] have reported a synergic effect between GNPs and hyperforin (Hyp) in the treatment of the EAE model. The data showed that free forms of GNP and Hyp significantly decreased the secretion of the pro-inflammatory cytokines (IL-17A, IFN-γ, and IL-6) and enhanced Th2/Treg cytokines (TGF-β, IL-10, and IL-4) compared to control mice. However, the Hyp-GNPs conjugates showed the most potent immunomodulatory effects via a significant increase in the IL-10, TGF-β, and IL-4 levels compared to both free-Hyp and free-GNPs. These immunosuppressive effects in EAE models are mediated by upregulating the express. Aboudzadeh et al. [151] demonstrated that positively charged chitosan-coated gold radionuclides (198GNPs@chitosan) showed a more cellular uptake and internalization a shorter time into MCF-7 cells compared to negatively charged citrate-stabilized GNPs and radionuclides (Gold-198 nanoparticles, or ^198^GNPs) and ^198^Au. It was supposed that the electrostatic interaction between GNP-chitosan with the cell membrane and endosomal escape resulted in more and faster internalization, which subsequently presented a higher dose of radioactivity to tumor cells, in turn, inducing a more effective cancer treatment.

Many other studies applied GNPs as efficient carrier delivery systems in clinical use, which showed promising results. For example, a study by Paciotti et al. [152] indicated a significant reduction in tumor volume and enhanced survival following uptaking functionalized GNPs with the combination of TNF and paclitaxel, which subsequently resulted in CYT-6091 human trials, a 27 nm GNP functionalized with recombinant human TNF-α (rhTNF) and PEG, which possess both immunomodulatory and cytotoxic effects [153]. In addition, it has been reported that functionalized GNPs with oxaliplatin [154] and or tamoxifen [155] were significantly internalized by human lung and breast cancer cells, respectively, subsequently demonstrating significantly increased cytotoxicity and much greater potency than the free drug. Therefore, GNPs open a new window for combination multi-therapeutic platforms, which by two different drugs at the optimized effective dosages and different acting mechanisms reduce the chances for the development of drug resistance, also improving therapy outcomes and reducing the systemic toxicity of drugs by targeted delivery and controlled drug release by various approaches such as laser light.

## 10. Application of GNPs in the Management of Autoimmune Disorders

### Application of GNPs for Treatment and Drug Delivery in Rheumatoid Arthritis

Rheumatoid arthritis is the most common chronic, immune-mediated inflammatory disease, affecting the joints and results in long-standing synovitis [156]. Although the exact pathogenesis of RA is still unclear, it is multifactorial, including genetic and environmental (dietary, infections, hormone) factors [157,158]. The prevalence of RA is approximately 0.5% to 1% of the population worldwide, associated with progressive disability, early death, and socioeconomic costs [159].

**Figure 2:**
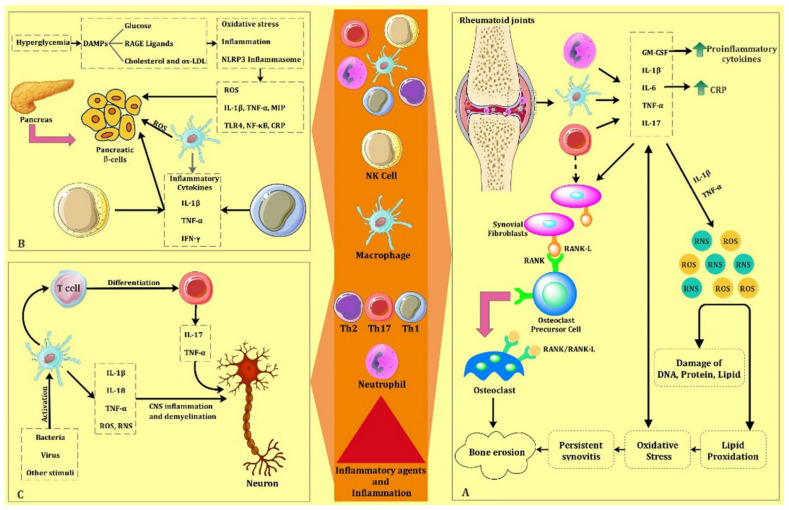
The role of immune cells and inflammatory cytokines in (**A**) rheumatoid arthritis, (**B**) diabetes, and (**C**) multiple sclerosis. A. Activated CD4 T-cells play an important role in RA pathogenesis and osteoclastogenesis by producing IL-17 cytokine, which subsequently induces RANKL on synovial fibroblasts and activates local inflammation, upregulating secretion of inflammatory cytokines such as TNF-α, IL-6, and IL-1 by synoviocytes, neutrophils, and macrophages. These inflammatory cytokines activate osteoclastogenesis by inducing RANKL on synovial fibroblasts or directly acting on osteoclast precursor cells. The increased cytokine production, especially TNF- and IL-1, stimulates synoviocytes, stress oxidation, and osteoclastogenesis. Moreover, Th17 cells also express RANKL on their cellular membrane, which partly contributes to the enhanced osteoclastogenesis. B. Various inflammatory cells involved in islet inflammation include macrophages as critical mediators via secreting TNF-a, IL-1b, and ROS. Autoreactive CD4 effector T-cells induce the inflammatory processes by release TNF-α, IL-1β, and IFN-γ cytokines, leading to the recruitment of CD8+T cells and macrophages. Moreover, NK, DCs, and NKT cells may have a partial role in the whole process via inducing pro-inflammatory cytokines. C. Activated macrophages by pathogens resulted in the activation of the NF-κB signaling pathway, subsequently inducing pro-inflammatory cytokines and free radical production. Moreover, the numbers of Th1 and Th17 cells and their cytokines such as IL-17, IL-1, IL-6, TNF-α, and IFN-γ are increased. Finally, the produced inflammatory and RNS/ROS mediators resulted in destroying the structure of the myelin sheath and neurons.

Inflammation plays a significant role in the progression of RA (Figure 2A). In this regard, the disease is characterized by synovial inflammation and increased synovial exudates, which leads to thickening of the synovium and swelling of the joint [158]. Pro-inflammatory cytokines such as TNFa, IL-6, and VEGF present an important role in exacerbating and maintaining joint inflammation [160,161]. Moreover, oxidative stress is a crucial event in RA pathophysiology, which plays a significant role in maintaining the inflammatory process and tissue damage within the inflamed synovium [162]. Oxygen-free radicals are involved in joint tissue damage of RA and experimentally induced arthritis [163]. A shift in the oxidant/antioxidant balance leads to lipid peroxidation. RA treatment has undergone many advances in recent years. The promising potential of GNPs in the treatment of inflammatory and autoimmune diseases has increased the interest in the study of the anti-inflammatory and antioxidant activity of GNPs in RA.

As mentioned above, gold nanoparticles are potent antioxidant and anti-inflammatory agents by quenching ROS, repressing the receptor activator of nuclear factor-κB ligand RANKL-induced osteoclast formation, which accounts for cartilage and bone erosion. Therefore, GNPs have been considered promising therapeutic agents due to their potential to modulate the main players of RA pathogenesis, including osteoclast, inflammatory cytokines, ROS, and VEGF mediators. Additionally, optical features [164,165] of GNPs lead to its use as contrast and nanoprobe agent to detect a target molecule and diagnose the progression of RA [73]. The application of gold compounds to treat RA dates back to Jacques Forestier’s [166] observations in the early 130s and for more than 50 years. Auranofin entered clinical trials in the 1980s. However, polymeric compounds for RA treatments are more effective than auranofin, although their use is associated with higher toxicity and adverse side effects [167]. Nevertheless, the use of cryotherapy has recently become less common for the management of RA.

Gold complexes (I) could potentially be an important tool in the management of RA. Chrysotherapy uses gold salts (a salt form of the metal element gold) for medical applications to treat diseases such as RA. Cryotherapy has been the mainstay of treatment for RA, with gold thioglucose (Solganol), sodium aurothiomalate (Myochrisine), sodium bis (thiosulfato) gold, and auranofin (Ridaura) among the most widely used therapeutic agents [168,169]. All these compounds contain gold in the +1 oxidation status.

The immuno-suppressive effect of gold (I) compounds has been described by inhibiting the production of pro-inflammatory cytokines such as TNF-α, IL-6, and IL-1β [170,171]. However, recent investigations have shown a much more complicated effect resulting in down or up-regulation of cytokine production, particularly when gold is associated with cell activators such as TNF-a or lipopolysaccharide [172,173,174]. Various mechanisms of gold (I) agents for anti-RA effects include (1) inhibiting cathepsins K and S; (2) repressing of hydrolytic enzymes such as β-glucuronidase and elastase [169], which play a role in the progression of RA (by aurothiomalate and auranofin) [175]; (3) targeting of thioredoxin reductase (TrxR) enzyme, which modulates cellular processes through the reduction of thioredoxin (Trx) [176,177]; (4) inhibition of leukocyte infiltration [177]; (5) changing macrophage activity [178]; and regulation of the adhesion of neutrophils [179]. Moreover, it also demonstrated that various gold (I) drugs’ metabolites modulate the immune system activity through direct binding to the T-cell receptors and then block antigen signaling [180], or may suppress T-cell activation via intervening with IL-2-mediated proliferative responses [181,182]. Additionally, they can indirectly bind to cysteine residues at the target antigen, preventing effective antigen-presenting to T-cells [180,183]. Moreover, B-cells are more sensitive to gold (I)-direct suppression effects than T-cells [184].

Despite these therapeutic uses, gold usually leads to undesirable immune reactions such as glomerulonephritis, nephropathy, thrombocytopenia induced by anti-platelet antibodies, lymphadenopathy, systemic reactions, and others in a maximum of one-third of patients [185,186,187,188]. The susceptibility to numerous side effects is related to genes in the MHC [188,189,190]. Natrium aurothiomalate (GSTM) is a useful disease-modifying antirheumatic drug but causes various immune-mediated adverse effects in some patients. Havarinasab et al. [191] showed that gold therapy with GSTM induces auto-antibodies against fibrillarin in genetically susceptible mice. However, despite the significant decline in their application, gold salts such as gold sodium thiomalate remain a useful agent in managing RA to date, and cryotherapy is still an important method in RA therapy. It has been reported that gold is a potent interactor with the immune system and attractive due to metallic gold’s ability to release large amounts of gold ions in the body [192]. However, long-term accumulation of gold salts in the body may lead to adverse or toxic effects.

Under in vivo conditions, the monovalent gold(I) drugs, without tightly bound to ligands, spontaneously dismutate to generate trivalent gold (AuIII) and zero-valent gold (Au0) forms [193]. Thus, it has been indicated that Au(0) is the active drug and is responsible for the antiarthritic property. At the same time, Au(III) causes side effects and toxicity observed during the RA therapy with gold(I) drugs [194,195]. Therefore, it is not surprising that various studies were assessed the anti-arthritic activities of GNPs (Au0) to avoid the side effects of RA treatment.

Some studies showed that GNP is a more potent and effective anti-arthritic agent and has significantly less toxicity than gold (I) drugs [163,177,196]. Brown et al. [163] have shown that although colloidal GNPs (Au0) with 27 ± 3 nm size were effective in inhibiting the progress of three different arthritis forms (including mycobacterium-, pristine-, and collagen-induced arthritis), sodium aurothiomalate (I) was only effective against mycobacterium-induced arthritis, common inflammatory arthritis characterized via active leukocytes producing ROS such as hypochlorite (ClO^−^) and H_2_O_2_. These results proved that GNPs are a more potent and effective anti-arthritic agent than sodium aurothiomalate (I) to treat rheumatoid arthritis [130] and suggested GNPs as a novel therapeutic tool for treating RA. In vitro studies of GNPs interacting with phagocytes have revealed their unique properties, such as their biocompatible, highly tissue-permeable, nonimmunogenic, and nontoxic features [197]. Hence, as an anti-arthritic agent, especially when administered intra-articularly, it is expected to be much less toxic than administered systemically conventional gold salts.

Numerous investigations focusing on cell systems presented data on how GNPs may exert their anti-inflammatory effects. For example, GNPs suppress activation of NF-κB [107] and subsequent inflammatory mediators and the production of nitrogen [148] and reactive oxygen species. However, precise mechanisms of their actions remain unclear because of a complex autoimmune response in the RA and the wide variety of biological targets within the body.

Collagen-induced arthritis (CIA) is a common animal model of RA characterized by similar pathophysiological changes to humans’ RA. An in vivo study by Tsai et al. [198] demonstrated that intra-articular administration of 13 nm GNPs improved the clinical course of the CIA rat model. GNPs presented antiangiogenic activities accompanied by inhibiting the proliferation and infiltration of inflammatory cells such as macrophages, reducing the NF-kB expression and subsequent inflammatory cytokines (TNF-α, IL-1β, and VEGF) and inflammation in the synovium, which led to the attenuation of arthritis. Another study by Leonaviciene et al. indicated that intra-articular injections of both 13 and 50 nm GNPs significantly reduced histopathological changes in the articular tissues and the development of chronic CIA, which mediated by reducing the MDA production and up-regulating the CAT activity, as well as decreasing inflammation [199]. In line with other studies [76,108], these data showed that GNPs acted as antioxidants and nontoxic agents.

Kirdaite et al. [200] observed that both intraarticular administrated 13 and 50 nm GNPs showed significant antioxidant activities by reducing malondialdehyde (MDA) production and significant increasing catalase activity, as an important antioxidant factor for the direct elimination of ROSs, without causing side effects on hematological parameters or internal organs in the experimental arthritis mice model. Moreover, 50 nm GNPs, when used as a prophylactic treatment in the primary stage of arthritis, significantly improve the formation of cartilage and excessive angiogenesis and suppress joint swelling more than with 13 nm GNPs. The erosive immune-mediated polyarthritis could be prevented and treated by GNPs in animals and humans [200]. These better anti-arthritic effects can be explained by the Rovais study that found 50 nm nanoparticles needed a shorter time for internalization than the smaller size of GNPs [151].

Angiogenesis and inflammatory cell recruitments into the synovium are initial histopathology responses in RA [201]. The most known strategy for the treatment of osteoarthritis (OA) and RA is the application of angiogenesis inhibitors, which interfere with the binding of VEGFA to its receptor (VEGFR) by various strategies and inhibit the subsequent VEGFA–VEGFR signaling (e.g., bevacizumab (Avastin^®^) [202], sorafenib, and sunitinib [203]). The VEGF-A targeting is a feasible anti-angiogenesis and anti-inflammatory therapeutic strategy for arthritis that could reduce the side effects. El-Ansary et al. [204] introduced a nano bioconjugation technique including anti-VEGFA-coated GNPs as a novel strategy for treating OA and RA diseases through inhibiting serum VEGF-A levels. The data have shown that anti-VEGFA-GNPs conjugate led to a reduction in serum VEGF-A level of OA and RA patients (in vitro). Therefore, this novel technique is considered a more promising therapeutic strategy for RA and OA patients than traditional therapies that are just a combination of drugs to reduce inflammation and help relieve symptoms.

Human RA synovial fluid contains elevated levels of bFGF, VEGF, and TNF-α [205]. The upregulation of VEGF-165 and its signaling pathway is a crucial factor in synovial angiogenesis. The VEGF165 could significantly induce activation and TNF production by mononuclear cells of synovial fluid in vitro [206]. Therefore, VEGF may present a mutual activation link between endothelial cells and macrophages/synoviocytes. It has been shown that 5 nm GNPs could inhibit VEGF 165-induced signaling and endothelial cell proliferation through interaction with the amines/sulfur present in the heparin-binding domain of VEGF165 and abrogating the VEGF association with its receptor [207]. Moreover, 13 nm GNPs have antiangiogenic effects due to their ability to bind to important angiogenic factors such as VEGF, both VEGF165 and VEGF121, and basic fibroblast growth factor (bFGF) in RA synovial fluid (SF), which could inhibit RA SF-induced endothelial cell migration and proliferation [198]. Therefore, GNPs may be a suitable therapeutic agent for treating arthritis and modulating various VEGF-dependent inflammatory diseases.

Although methotrexate (MTX) is one of the first-line anti-rheumatologic agents due to its good therapeutic efficacy, its long-term administration may induce serious side effects [208]. Interestingly, a study by Lima et al. reported that MTX attachment to GNPs enhanced its efficacy and greatly improved the MTX release rate under acidic conditions or at photo-thermal temperature (42 °C). Furthermore, the data showed this co-incorporation significantly suppressed the production of pro-inflammatory cytokines (TNF-α, IL-6, and IL-1β) compared to MTX and GNPs treatments alone, which generated a pronounced anti-inflammatory effect and improved RA [148]. Therefore, they proposed the MTX-loaded multifunctional GNPs as a promising theranostic platform for diagnosing and treating RA, which presents a highly effective targeted chemo-photothermal therapy.

The IL-6 is another important pleiotropic cytokine in RA, highly expressed in RA patients’ serum and synovial fluid levels [156]. The IL-6 shows a significant role in the hepatic acute phase response via high activation of neutrophils, monocytes, B- and T-cells [209]. Therefore, the inhibition of this cytokine is the second common purpose of RA treatments with biological drugs. The novel platform of hyaluronate (HA)/GNP–protein complex could be applied as a delivery carrier for numerous therapeutic targets [210,211]. Lee et al. have shown that the dual-targeted HA-GNP/TCZ complex bound to both the IL-6 receptor and VEGF. In this regard, GNPs were applied as a drug carrier attached to VEGF and resulted in antiangiogenic effects. In contrast, the immunosuppressive TCZ drug, a monoclonal antibody against the IL-6 receptor, interfered with IL-6 in the pathogenesis of RA, and HA is also widely used for lubrication and cartilage protection. These findings showed GNP’s antiangiogenic effects on the HUVECs proliferation via binding to VEGF [211].

Nevertheless, a study by James et al. [212] reported contradictory results with mentioned studies. They revealed that gold compounds such as auranofin had a pronounced ability to decrease the production of several inflammatory mediators such as reactive NO, ROS, and TNFα by LPS-induced macrophages compared to GNPs. However, although GNPs had little or no significant effect on LPS-induced production of ROS, NO, and IL-10 or showed a low inhibitory effect on TNFα production, GNPs had lower cytotoxicity than the gold complexes, despite more accumulating into the cells. Some possible reasons for the general absence of the biological activities by the GNPs were explained, including that the mechanisms involved in the inflammation that werenot investigated here, the in vitro conditions might not have been optimal, or the GNP usage may not produce any substantial changes to macrophage function due to the absence of mechanisms available in vivo. Therefore, it is plausible that applying higher doses of GNPs might lead to more significant impacts.

**Figure 3:**
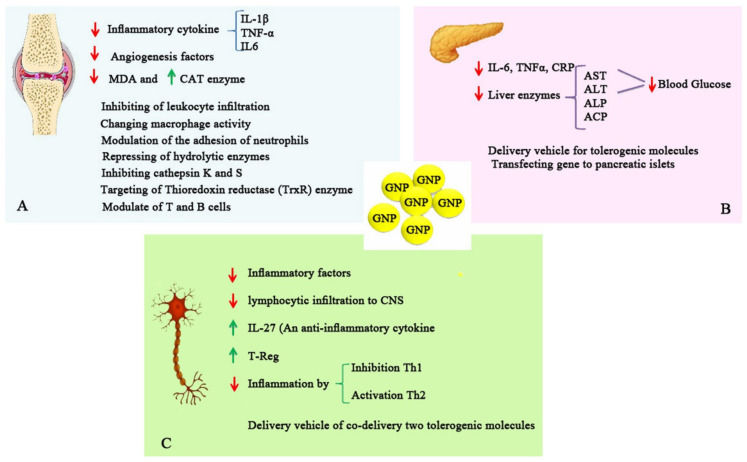
Simplified illustration of immunomodulatory effects of GNPs and their therapeutic applications in autoimmune diseases, including (**A**) rheumatoid arthritis, GNPs exert anti-inflammatory and anti-oxidant effects; (**B**) diabetes, anti-inflammatory and anti-oxidant effects, and their application as a gene delivery carrier; and (**C**) multiple sclerosis; anti-inflammatory and anti-oxidant effects accompanied by application as a delivery system to inducing tolerance.

The studies mentioned above highlight the great potential of GNPs as a candidate for RA treatment. Nevertheless, further studies are still needed to evaluate how much GNPs may exert their anti-arthritic effects and their effectiveness compared to traditional gold (I) drugs and determine the possible mechanisms of their antiarthritic activities. Nevertheless, some possible activities of GNPs in RA are shown in Figure 3A.

## 11. Application of GNPs in Diabetes and T1DM

Diabetes includes a group of chronic metabolic disorders identified by hyperglycemia resulting from disrupting insulin secretion or action. The most common types include type 1 diabetes (T1D) and type 2 diabetes. T1D is the autoimmune destruction of insulin-producing pancreatic β-cells by the immune system, which leads to absolute insulin deficiency. In contrast, other factors such as insulin resistance come into play in type 2 diabetes. Insulin is the mainstay of treatment for patients with T1DM, which can result in hypoglycemia and weight gain [110]. Therefore, an efficient and economic molecular therapy that promotes the treatment of diabetes by controlling the hyperglycemia-induced oxidative stress, disrupting several metabolic pathways and thus preventing complications is a crucial research subject.

Several studies have reported alternative strategies to reduce the frequency of insulin injections. The use of carriers is the most promising strategy, which results in an increased duration of action of injected insulin. Among various carriers, organic carriers and metal nanoparticles have been considered potential carriers to enhance drug delivery due to their controlled release capabilities and biocompatibility [213,214,215,216,217]. On the other hand, among various pathogenic pathways, the high concentration of glucose results in producing ROS and eventually causes diabetic complications and metabolic abnormalities [218]. Furthermore, increased oxidative stress can occur due to the excessive production of ROS and its inefficient scavenging [219]. Collectively, oxidative stress plays a principal role in diabetes progression, resulting in various diabetes complications [220,221] (Figure 2B). Therefore, using a biological antioxidant agent will be an effective strategy to suppress the progression of diabetes caused by oxidative stress during hyperglycemia.

The promising potential of GNPs in the treatment of various inflammatory and autoimmune disorders led to further interest in examining antioxidant, anti-inflammatory, and anti-hyperglycemic activity accompanied by their capability for extending the duration of insulin action in treating diabetes mellitus. Barath Mani Kanth et al. [110] reported that GNPs showed anti-hyperglycemic and anti-oxidative activities in diabetic mice via adjusting the ROS production at hyperglycemic conditions and improving the antioxidant enzyme systems scavenging free radicals. Moreover, it has been shown that GNPs show a nontoxic nature and also protect different organs without causing harmful effects. Therefore, GNPs exerted sustainable management in the progress of the disease.

It has previously been reported that the potential of GNPs as a carrier of trans-mucosal insulin delivery [216] in the treatment of diabetes mellitus [217] leads to improved pharmacodynamic activity. However, for the first time, a study by Liu et al. reported an approach to producing insulin-stabilized gold nanoclusters, which showed excellent biocompatibility and retained insulin bioactivity in a mouse model [222]. In parallel with this data, Shilo et al. produced insulin-coated GNPs, which prevented rapid insulin degradation that led to controlled and adaptable bio-activity. Hence, the immobilized insulin on the GNP surface is active and is even longer than free-form insulin [223].

Lee et al. applied dextran-encapsulated GNPs as insulin carriers to prolong insulin activity. The data showed that GNP@Dextran–insulin compounds are proper carriers that could prolong insulin activity three times more than the free form of insulin [224]. Therefore, GNPs can be potentially applied as the carrier to extend insulin activity for reducing the frequency of insulin injection in diabetes mellitus. Kumari et al. [225] showed that the GNPs–insulin conjugate improved various parameters such as antioxidants, blood glucose levels, various liver and kidney parameters, body weight, and lipid profile on STZ-induced diabetic rats. TNF-α exhibited a complicated intersection with T1DM and caused the beta-cell injury that was followed by chronic hyperglycemia. Both hyperglycemia and TNF-α lead to impaired insulin signaling. It has been indicated that this cytokine shows a dual function in autoimmune diabetes according to exact timings where the autoimmune process in T1DM is determined through inflammatory factors [226]. Therefore, the development of diabetes could reduce by administering exogenous TNF-α via an effect on the decreased production of endogenous TNF-α in NOD mice. On the other hand, increased IL-6 and TNF-α serum levels represent the insulin resistance state. Moreover, high levels of these cytokines accompanied by CRP were shown in the newly diagnosed T1DM in children [227]. Moreover, TNF-α can lead to insulin resistance related to obesity, which has a significant role in atherosclerosis [228]. Several studies showed the GNPs role in reducing inflammatory cytokines levels and ameliorating type 1 diabetes via suppressing inflammation [92,229].

A study by Karthick et al. [229] reported that GNPs therapy led to a significant TNF-α, IL-6, and CRP reduction in the diabetic model, which subsequently reduced blood glucose and increased serum insulin levels. In addition, it has been shown that 10 nm GNPs control hyperglycemia in diabetic rats via regulating blood glucose levels, insulin resistance, pro-inflammatory mediators (CRP, IL-6 and TNF-α), and liver enzymes [92]. These data proposed the possible role of GNPs as a cost-effective therapeutic modality in the treatment and management of T1DM and its complications. Barath Mani Kanth et al. [110] reported that GNPs showed anti-hyperglycemic and anti-oxidative activities in diabetic mice via adjusting the ROS production at hyperglycemic conditions and improving the antioxidant enzyme systems’ scavenging free radicals. Moreover, it has been shown that GNPs show a nontoxic nature and also protect different organs without causing harmful effects. Therefore, GNPs exerted sustainable management in the progress of the disease. In contrast, Selim et al. [230] for the first time demonstrate that GNPs significantly intensified antioxidant production in STZ-induced diabetic rats, a known model for T1DM. Additionally, they stated no difference between the blood glucose level in comparison with control groups. However, they illustrated a significant reduction in uric acid and creatine levels compared with the control group. On the other hand, the FoxP3+ Treg cell population is an important subset for T1D pathogenesis [231]. The alterations of numbers and the function of pancreatic Tregs have been reported in recent-onset T1D patients [232]. The administration of ex vivo tolerogenic DCs or Treg cells can be considered as a potential therapy for T1D, although cell-based therapy approaches are difficult processes in clinical practice [233,234]. Another study by Yeste et al. [235] used the engineered GNPs as a delivery system for two tolerogenic molecules, which subsequently induced tolerogenic DCs phenotype and promote Treg cell production via the inhibition of pro-inflammatory cytokine production and NF-kB activation. Collectively, these findings indicated that GNP-based therapies might be a potential tool to stimulate tolerance in T1DM and other ADs.

Another effective treatment for type 1 diabetes is pancreatic islets transplantation. Altering their gene expression profile could improve engraftment and its survival and prolong the islet graft lifespan [236]. Although the application of viral and non-viral vectors to genetic material transfer islands is an encouraging approach to gene-regulating, safety and efficiency deficiencies led to an interest in designing new transfusion strategies. Polyvalent GNP-DNA conjugates have unique properties, which densely functionalize with DNA oligonucleotides [237]. Therefore, they can be the new tool for transfection and gene regulating that can enter cells with high efficiency and no evidence of toxicity [238]. Jonathan et al. have shown that GNP-DNA conjugates could efficiently transfect into pancreatic islets without altering the cellular viability or functionality and could regulate the expression of targeted genes. Overall, these conjugates may represent the next generation of nucleic acid-based therapeutic agents to improve the transplantation of pancreatic islets, survival, and long-term function [236].

Therefore, GNPs has opened up the way for a novel therapeutic agent for the management of diabetes and its complications via (1) enhancing the antioxidant defense enzymes and performing a maintained control across the hyperglycemic condition and (2) delivery and prolonging insulin activity for reducing the frequency of insulin injection (Figure 3B). Nevertheless, further studies are required to assess mechanisms and the downstream pathways of GNPs that influence the antioxidant systems and their reverse effect over hyperglycemic states to offer future therapeutic applications of GNPs in diabetes mellitus.

## 12. Application of GNPs in Multiple Sclerosis

Multiple sclerosis (MS) is a chronic inflammatory demyelinating and neurodegenerative disorder of the central nervous system (CNS), resulting in irreversible brain and spinal cord injury. It is the leading cause of severe physical and neurological disability in adults, which affects more than 2.5 million people worldwide. Although the etiology of MS remains unclear, it is probably the result of a complex interaction of genetic, environmental exposure, and lifestyle factors. In addition, the abnormal immune response to self-myelin antigens and the immunoregulatory system defects play a significant role in disease pathogenesis [239].

It is considered that myelin antigen-specific T-cells are essential in initiating and organizing inflammatory cascade in CNS [240]. Both MS and experimental autoimmune encephalomyelitis (EAE), the best experimental model for human MS, have long been known as a Th1-mediated autoimmune disorder [241]. Nevertheless, the immune dysregulation of the Th1 responses and Th1/Th2 paradigm cannot fully explain the immunopathogenic mechanisms underlying the MS/EAE pathogenesis. Numerous studies have shown the crucial role of Th17 cells in inflammatory and autoimmune disorders of the CNS, particularly in the early stages of the disease. It has been reported that the transferred myelin-specific Th17 cells to the CNS resulted in secreting IL-17A, which subsequently attract the immune cells into the CNS via inducing the production of chemokines and eventually start and preserving the inflammatory cascade. The role of Th1 cells in pathogenesis is probably more pronounced in the later stages of the disease. Therefore, the Th17/Th1 paradigm is the current perspective about T-helper cells role in MS and EAE pathogenesis. [240]. MS patients have increased numbers of Th1 and Th17 cells accompanied by their cytokines such as IL-17, IL-1, IL-6, TNF-α, and IFN-γ [242,243,244] (Figure 2C).

Moreover, the Tregs of these patients have various abnormalities. In addition to the aberrant pathogenic T-cells, dysfunctional or impaired maturation of Treg cells also can promote EAE. Tregs have been shown to arrest the development of several experimental models of AD. Furthermore, they may have protective effects against the EAE progression via anti-inflammatory cytokines [245]. Thus, the induction of antigen-specific tolerance is considered a promising approach to treating MS and other autoimmune disorders. Despite numerous studies, the optimal therapeutic agent for MS is still elusive. MS patients have increased numbers of Th1 and Th17 cells accompanied by their cytokines such as IL-17, IL-1, IL-6, TNF-α, and IFN-γ [242,243,244] (Figure 2C). Moreover, the Tregs of these patients have various abnormalities [246,247]. Despite numerous studies, the optimal therapeutic agent for MS has is still elusive. Nanoparticles such as silver, iron oxide, and gold can increase survival, differentiation, and neuronal growth. Among NPs, GNPs have widely been investigated, and it has been reported that delivering electrical stimulation leads to increased PC-12 cell differentiation and enhanced electrical excitability of neuronal cells [248]. Moreover, it showed that GNPs administration into the middle cerebral artery occlusion model led to anti-inflammatory and antiapoptotic effects. These anti-inflammatory and antiapoptotic effects included an increase in the anti-inflammatory cytokines production and the regulation of apoptotic molecules, which resulted in improving neurological defects and decreasing infarct volumes [249].

Another study by Papastefanaki et al. showed that 14 and 40 nm diameter PEG–GNPs reduced inflammatory responses and microglia responses, increased motor neuron survival and axon myelination, and improved overall clinical symptoms [250].

Aghaie et al. [251] investigated the effects of GNPs and PEG separately in the EAE model. The data showed that 25 nm GNPs significantly reduced the clinical symptoms in the EAE model via significant increased anti-inflammatory cytokine levels such as IL-27 and the reduction in lymphocytic infiltration and demyelination of the CNS in the GNPs-treated groups. Moreover, the PEG-treated group significantly reduced the pro-inflammatory cytokine (IL-23) and increased the half-life of therapeutic agents and the drug efficacy. Therefore, GNPs and PEG were introduced as novel promising therapeutic agents to improve clinical symptoms of MS and a therapeutic potential of PEGylated GNPs was proposed for the treatment of MS due to the intrinsic immunomodulatory and therapeutic properties of GNPs accompanied by the PEG polymer.

As mentioned above, Nosratabadi et al. [150] investigated the effects of hyperforin (Hyp), free and conjugated with GNPs, in the EAE model. They reported that free forms of GNP and Hyp showed the immunosuppressive mechanisms and significantly reduced inflammatory cells infiltration into the CNS, raised Treg and Th2 cells, and reduced Th1 and Th17 cells in EAE models. Interestingly, Hyp-GNPs treatments with various doses were superior in reducing disease severity, via significant impact on the IL-10, TGF-β, and IL-4 levels, compared to free forms of Hyp and GNP. Therefore, they proposed the GNP–hyperforin conjugate as a novel nano pharmaceutical with synergistic immunomodulatory characteristics on inducing Treg and Th2 expansion and inhibiting Th17 and Th1 differentiation, which could delay or suppress the progression of EAE.

Yeste et al. [252] applied 60 nm GNPs as a delivery vehicle for co-delivery of two tolerogenic molecules (include MOG 35–55 and ITE(2-(1′ H-indole-3′-carbonyl)-thiazole-4-carboxylic acid methyl ester)) to an expansion of Tregs by DCs. They reported that GNP-mediated co-delivery suppressed EAEs via the development of tolerogenic DCs and promoted the differentiation of Tregs in vitro. Moreover, GNPs carrying ITE and MOG35–55 expanded the FoxP3+ Treg compartment and suppressed the EAE development (Figure 3C). Thus, GNPs are potential new tools to induce functional Tregs in autoimmune disorders. Therefore, the attachment of therapeutic agents on the GNPs surface via increasing the therapeutic properties of drugs and their delivery to specific targets accompanied with the intrinsic therapeutic properties of GNPs can be considered a promising choice to modify agents to the management of MS development.

## 13. Application of GNPs in Other Autoimmune Diseases

TNF-α and IL-12 are important pathogenic factors in the development of psoriasis [253]. Hence, the inhibition of TNF-α- and IL-12-producing pro-inflammatory macrophage in psoriasis lesions through topical medication delivery may present a safe alternative therapy. Crisan et al. [254] reported that GNPs complexed with Cornus mas (GNPs-CM) modulated inflammation in psoriasis at the cellular and molecular levels. They demonstrated that the incubation of macrophages with GNPs significantly reduced the production of IL-12, TNF-α, and NO through suppressing NF-κB activation and substantially reduced inflammatory macrophages and TNF-α and IL-12 production in human psoriasis plaques. Moreover, this study showed for the first time that GNPs efficiently prevented the activation of macrophages in both in vitro and in vivo conditions, which finally resulted in disease resolution. Hence, GNPs may represent a safe and effective agent for modulating the inflammatory condition in psoriasis and usually reduces the adverse effects of systemic treatments.

A study by Hussein et al. [131] investigated the treatment potential of intravenously administrated GNPs at various sizes (16–25 nm) and doses (40 and 400 μg/kg) in experimental colitis. These data showed that GNP treatments suppressed the inflammatory response by decreasing the pro-inflammatory cytokine levels, including TNF-α and IL-6, which eventually reduced the colon inflammation and diminished the oxidative stress markers. Moreover, it reported that the high GNP concentration (400 μg/kg) resulted in the most significant therapeutic effects in colitis rat models. Thus, it suggested that GNPs may have therapeutic potential for colitis treatment without noticeable adverse effects.

Barreto et al. [124] demonstrated that intranasally administrated GNPs in the atopic asthma model resulted in significant downregulating of pro-inflammatory cytokines (IL-1, IL-13, IL-6, IL-5, and IL-4), reactive oxygen species, and chemokines (eotaxin-1 and eotaxin-2), which was associated with inhibiting the accumulation of the inflammatory cells in lung tissue. The data showed that downregulating the oxidative stress levels is probably the related mechanism for the immunomodulatory effects of GNPs.

## 14. Gold Nanoparticles as Potential Diagnostic Devices for Autoimmune Diseases

At present, the diagnosis of ADs is mainly based on the physician evaluation accompanied by laboratory tests. However, these tests have several deficiencies, including low sensitivity, being expensive, and the need for advanced equipment. In addition, due to the low sensitivity of these tests, they cannot detect early molecular events, so often, the disease is diagnosed too late, at a time when irreversible tissue damage has already occurred [255,256].

In recent years, biosensors have become more acceptable due to their several advantages, including increased sensitivity due to lower sample volume, electrochemical detection, and less time required for analysis. Numerous detection methods, including colorimetric, impedimetric, chemiresistive, and electrochemical-based methods, have been described [72,256,257,258]. The electrochemical biosensor is an attractive alternative to other analytical means by providing fast, sensitive, specific, and cost-effective methods, widely applied to detect numerous biological molecules [259]. Various electrochemical sensors have been developed to diagnose autoimmune diseases.

Among them, metal nanoparticles, especially gold and silver, have attracted a lot of attention and are employed to develop immuno-sensors to autoimmune disorders (Table 2). Due to their redox features, these NPs generally conjugated with the detection Abs, metal-based nanoparticles can produce signals in specific situations, such as in acidic states for GNPs [260]. Moreover, GNPs can be used as labels with electrocatalyst, facilitating the redox reaction of a redox-active compound added to the system. The electrochemical platform consisted of GNPs-functionalized carbon screen-printed electrodes on transglutaminase immobilized, which capture anti-tissue transglutaminase antibodies (anti-tTG), generating an amperometric signal via a secondary Ab labeled with alkaline phosphatase (AP). It was suitable for celiac disease diagnostics [261]. Fernández et al. applied the gold electrode as an immobilization surface for glucopeptide antigen. They evaluated the specific binding of serum antibodies to the immobilized antigen, which showed that it led to an enhanced result, and a detection limit was obtained. Therefore, these biosensors could potentially be used to determine the prognosis of MS [74].

Among several biosensors using different electrode architectures to detect matrix metalloproteinase-7 (MMSP-7), the best limit was obtained for MMP-7 by Kou et al. [271]. They used a peptide and an ssDNA-S1-modified platinum nanoparticle (P1-PtNPs-S1) as recognition nanoprobes, immobilized on GNP-modified glassy carbon electrodes. Hence, it offered a promising avenue for the detection of other proteases.

Neves et al. [265] developed a transglutaminase electrochemical immunosensor to detect celiac disease using cyclic voltammetry. The immunosensor consists of screen-printed carbon electrodes (SPCE) nanostructure with carbon-nanotubes and GNPs, as the transducer surface. The tTG, as a biorecognition element, is immobilized on the transducer surface to detect TG autoantibodies in serum samples. The carbon–metal nanoparticle conjugation was excellent for amplifying immunological interactions. Therefore, this method, via a combination of the benefits of powerful antibody–antigen interaction and the sensitivity of the electrochemical techniques, could create a new disposable electrochemical immunosensor for detecting anti-tTG IgA and IgG autoantibodies. Compared to the ELISA test, the results from the electrochemical immunosensor were qualitatively matched (i.e., positive or negative) since the diagnosis often relied more upon qualitative results. They suggested electrochemical immunoassay is an excellent point-of-care diagnostic tool for detecting celiac disease-specific anti-tTG autoantibodies in sera samples, which can be a great alternative to the conventional optical screening assays. Gupta et al. [72] reported that a transglutaminase-based nanosensor consists of the sensor PAMAM/GQD nanohybrid modified on GNP embedded in multiwalled carbon nanotubes (MWCNT)-based immunosensor, which is highly specific to anti-tTG and revealed a negligible response to non-specific serum proteins. The sensor’s sensitivity was about 1297.1 pg, and a detection limit was found at 0.1 fg per 6 µL. In another study, Kaur et al. [266] developed a screening test based on the 20 nm GNPs functionalized with a sequence of the gliadin-derived peptide, which triggers the CD. They demonstrated that this functionalized GNP with a novel peptide-based assay is helpful for pre-selecting CD, especially in high-risk pediatric populations confirmed by mucosal biopsy.

As mentioned, early diagnosis of ADs results in reducing the frequency of premature morbidity and mortality. Almost all ADs possess their own set of biomarkers such as proteins, peptides, or Abs, which are in body fluids. Therefore, an ideal care device can detect the ADs markers before any symptoms’ advent. Many attempts are already stated in several studies, and relevant reviews were considered for electrochemical biosensors, representing viable alternatives for developing point of care devices. The developed assay has high efficiency levels and is approximately more economical; the assay format has the potential to be adapted as a point of care test (POCT) that would be useful in an exclusion diagnostic strategy. A positive result could strengthen the possibility of a CD that an intestinal biopsy can confirm.

## 15. Conclusions

The proper design of nanoparticles is a fundamental requirement for the more efficient application of nanotechnology. Many fundamental studies must evaluate the interaction of nanostructures with biological systems for providing more guidance. In this way, so many studies have started to design multifunctional NPs. Applying NPs such as GNPs to treat inflammatory and autoimmune diseases will offer innovative solutions for efficacy improvement of current immunosuppressive treatments, which will help overcome the side effects of these therapies. It showed that colloidal GNPs could be utilized in applying nanotechnology in medicine to treat and detect ADs such as RA, T1DM, and MS.

The GNPs represent a new generation of drug delivery systems with high drug-loading capacity that can employ a wide range of materials. Furthermore, as mentioned above, GNPs are potent antioxidant and anti-inflammatory agents, resulting in quenching ROS, reducing RANKL-induced osteoclast formation and inflammatory cytokines (IL-1, IL-6, and TNF-a) VEGF, which are the main contributors to the RA pathogenesis. Therefore, GNPs can be considered as a potential novel therapeutic agent for the treatment of RA.

Moreover, hyperglycemia in diabetes could be controlled by GNPs via improving blood glucose levels, insulin resistance, and liver enzymes, reducing proinflammatory cytokines (include TNF-α, IL-6, and CRP), and enhancing the antioxidant defense enzymes. Moreover, GNPs via delivery and prolonging the insulin action could reduce the frequency of insulin injections. In MS, GNPs can increase differentiation, survival, and the growth of neuronal, anti-inflammatory, and anti-apoptotic effects, improving neurological defects. In addition, they decrease infarction volumes, eventually improving overall clinical symptoms. Moreover, GNPs, as a delivery vehicle of tolerogenic molecules, could promote the generation of Tregs by DCs cells.

This review shows that maximal intracellular uptake and responses to GNPs vary according to their characteristics such as size, surface charge, concentration, and the route of administration and duration of exposure, which need to be considered in nanosystem design.

It is suggested that GNPs can be a promising delivery system for therapeutic and targeting agents for diagnostic and therapeutic purposes in ADs. Therefore, GNPs open a window for designing next-generation multifunctional nanosystems platforms that can easily be functionalized with therapeutic agents or drugs. Furthermore, the capaccellular controlled release of drugs resulted in significant potential for enhancing active targeted delivery and efficiency drugs while reducing effects. Collectively, although preclinical studies suggested GNPs as an efficient potential alternative for treating ADs, several challenges still need to be overcome before this technology be developed further as a new therapeutic approach in clinical use. Therefore, further comprehensive studies for evaluating body distribution, stability, in vivo safety, and the exact immunomodulatory effects of GNPs could be the beginning of a major shift toward novel treatments for the clinical settings.

## Figures and Tables

**Table 1:** The effects of bare GNPs on the production of inflammatory and oxidant parameters.

Characterization of GNP	Animal Model/Cell Line	Mechanism of Actions	Main Effect			Ref.
			Inflammatory	Anti-Inflammatory	Anti-Oxidant	
5, 15, 20, and 35 nm GNPs	C57BL/6 male mice—THP-1 cells	-Decreased TNF-a		✔		[96]
		-Decreased HIF-1				
		-Decreased NF-kB				
		-Down-regulation of IL-1β-induced inflammatory by reducing NF-kB.				
		-The 5 nm AuNPs completely blocked the inflammatory process, 15 nm AuNPs were less effective, and 35 nm AuNPs did not display a statistically significant effect.				
35 mm	Rat	-Decreased IL-1b		✔		[101]
		-Decreased TNF-a				
10 nm	Rat	-Decreased IL-1b		✔		[91]
		-Decreased TNF-a				
50 nm	RAW 264.7 macrophages	-Decreased IL-1b		✔	✔	[95]
		-Decreased ROS				
		-Reduced interleukin (IL)-17 and TNFα triggered by LPS				
15 nm	Mice	-Reduced interleukin (IL)-17a		✔		[68]
		-Reduced neutrophil recruitment				
		-Increased IL-10 levels				
6.3 nm	Mice	-Reduced the levels of IL-1, IL-5, and IL-6 in the BAL		✔	✔	[124]
		-Reduced IL-4, IL-5, IL-6, IL-13, eotaxin-1, and eotaxin-2 in lung tissue				
		-Inhibited inflammatory infiltration in the airways				
		-Significant reduction in the levels of malondialdehyde (MDA)				
10 and 50 nm	Rat Liver	-Both sizes significantly transient increase cytokine gene expression include TNF-α, IL-6, and IL-1β	✔			[81]
		-The GNPs with 50 nm size induced more severe inflammatory responses compared to smaller GNPs.				
3, 11, 16, 30, and 40 nm	RAW264.7 (ATCC, TIB-71) SV40-transformed endothelial cells SVEC4-10 7 (ATCC, CRL-2181) and the murine mesenchymal stem cell line C3H10T1/2 (ATCC, CCL-226)	-Macrophage production of the monocyte chemoattractant RANTES/CCL5 depended on GNP size, i.e., GNP 11 nm significantly decreased CCL5 secretion while GNP 16 nm had the opposite effect.	✔			[125]
		-Enhanced TNFα secretion				
		-Did not induce IL-10 secretion				
10, 30, 50, and 80 nm	BALB/c mice	-50 nm GNP significantly induced the M1 macrophage phenotype.	✔			[123]
		-Increased IL-b, IL-6, and TNF-a in 50 nm nanospheres treatment				
		-50 nm GNP via activation of the NF-κB signal pathway led to SAA activation				
50–250 nm	Wistar-derived rats	-Increased of in IL-6 and TNF-a 250 nm single GNPs	✔			[118]
		-Significant increase in immune cells, especially macrophages				
		-Increased MCP-1 and MIP-2				
		-Increased TNF-α and IL-6 levels after treatment with 250 nm single GNPss				
		-Increased neutrophils after 24 h along with single 250 nm particles				
10–15 nm	RAW264.7 cells	-Blocked the activation		✔	✔	[107]
		-Inhibitory effects on IFN-b mRNA expression				
		-Attenuate nitric oxide levels				
20 nm	Rat	-Decreased IL-1β		✔	✔	[90]
		-Downregulated mRNA expression of iNOS, COX-2, TNF-α, and NF-kB				
Up to 5 nm	Mice	-All cytokines were unaffected along with intermediate concentrations (2.5–5 ppm)		✔		[126]
		TNF-α and IL-1β significantly decreased along with the highest concentration (10 ppm) but stimulated IL-6.				
		Production of TNF-α and IL-2 was decreased along with low concentrations but stimulated IL-1α				
25–50 nm	NHDF and NHEK	-Decreased TNF-a		✔ + antiangiogenic activity		[127]
		-Decreased IL-6				
		-Decreased of IL-2 levels				
		-Decreased proteins involved in angiogenesis such as VEGF and bFGF.				
20 nm	RAW264.7 cells	-Decreased gene expression of MMP-2/-9, CX3CL-1, CCL-8, CX3CL-10, ICAM, IL-1α, and TNF-α in a dose-dependent manner		✔	✔	[89]
		-Inhibited of NF-κB pathway via ERK1/2MAPK/Akt/tuber in-mTOR kinases interference, which resulted in reducing oxidative-nitrosative stress				
Auranofin		-Blocked IL-6		✔		[128]
		-Blockaded of JAK1/STAT3 signalling.				
Au-S = 2.81 Au-M = 5.52 Au-L = 38.05	cell culture	-GNPs (especially those with a smaller diameter) up-regulate the expressions of pro-inflammatory genes		✔		[113]
		-IL-1, IL-6, and TNF-a.				
		-Expressions of proinflammatory genes decreased with the increased size of AuNPs				
10 and 50 nm	Rat	-Increased cytokines gene expression by both sizes of GNPs (10 and 50 nm) in the liver				[120]
		-The GNPs with 50 nm size induced severe inflammatory response compared with smaller GNP size				
		-The GNPs do not have any effect on IL-1β in the kidney				
		-The GNPs with 10 nm size do not have any effect on TNF-𝛼 and IL-6 gene expression				
		-The GNPs with 50 nm size significantly increase expression of IL-6 and TNF-𝛼 in the kidneys of rats				
10–50 nm	Leukemic cell lines (T-lymphocytic Jurkat and monocytic U937 cells)	-Stimulated TNF-α production		✔		[129]
		-Inhibited interleukin-6				
		-Inhibited interleukin-2 production				
5.5 nm	Cell culture (HUVECs; VECs)	-Reduced TNF-a		✔	✔	[93]
		-Reduced monocyte adhesion to VECs in vitro and arterial				
		-Reduced NF-kB				
		-Reduced ROS				
25 nm	Wistar rats	-Decreased TNF-a		✔	✔	[130]
		-Decreased IL-6				
		-Decreased SOD and Catalase (CAT) activity				
		-Decreased superoxide and Nitrite levels				
16–25 nm	Wistar rats	-Decreased TNF-α levels		✔	✔	[131]
		-Decreased IL-6 levels				
		-Significant decrease in antioxidant markers such as -GSH, SOD and CAT in the colon.				
30–40 nm	Rat/in vitro	-Downregulation of TNF-a		✔		[132]
		-Controlled IL-6 secretion				
		-Upregulation IL-10				

**Table 2:** Gold nanoparticle-based applications for diagnosis and monitoring of autoimmune diseases.

Disease	Electrode Architecture	Target	Label	Detection Method	Sample	Ref
**Celiac Disease**	GNPs/SAM-GCE	IgA anti-tTG	AP	CPV	Serum from patients	[262]
		IgB anti-tTG				
	CNTs/GNPs-SPE	IgA anti-tTG	AP	CV	Serum from patients	[263]
		IgB anti-tTG				
	Au/SAM-GCE	IgA anti-tTG	HRP	CV	Serum from patients	[264]
		IgB anti-tTG				
	Screen-printed carbon electrodes (SPCE) nanostructure with carbon-nanotubes and GNPs	IgA and IgG type anti-tTG	AP	CV	Serum from patients	[265]
	GNP-Peptide-AGA	anti-gliadin antibody			Spiked samples Serum from patients	[266]
	GQD/PAMAM/GNP/MWCNT	IgA anti-tTG		DPV with redox probe	Human serum	[72]
	Poly (sodium-4-styrensulfonic acid)-gold SPE	Anti-tTG	POD	EIS	Serum from patients	[267]
	Gold electrodes with carboxylic-ended bipodal alkanethio	AGA	HRP	Chronoamperometry	Serum from patients	[268]
**Rheumatoid Arthritis**	GNPs-NTiP-Thi-gold electrode	MIF		DPV with redox probe	Serum from patients	[264]
**Non-Specific Biomarkers**	Electroplating gold onto a disposable printed circuit board electrode	IL-12		EIS	Spiked serum	[269]
	GNPs-PDA-GO	HIgG	AgNPs/carbon nanocomposite/benzoquinon		Spiked serum	[270]
**Multiple Sclerosis**	Gold sensor chip	Glucopeptide CSF114(Glc) antigen		SPR detection system	Serum	[74]

IgB, immunoglobulin B; IgA, immunoglobulin A; IL-12, interleukin 12; MIF, Mamacrophageigration inhibitory factor; POD, peroxidase; DPV, differential pulse voltammetry; AP, alkaline phosphatase; anti-tTG, anti-transglutaminase Abs; AGA, anti-gliadin Abs; HIgG, human immunoglobulin G; CV, cyclic voltammetry; HRP, horseradish peroxidase; CNC, carbon nanocomposite.
